# Comparison of custom-designed oral stents in radiotherapy for head and neck malignancies

**DOI:** 10.3389/fbioe.2025.1544105

**Published:** 2025-06-13

**Authors:** Shihang Li, Guobao Pang, Rong Li, Qinying Shi, Yannan Xu, Ying Lu, Jianbo Song

**Affiliations:** ^1^ Third Hospital of Shanxi Medical University, Shanxi Bethune Hospital, Shanxi Academy of Medical Sciences, Tongji Shanxi Hospital, Taiyuan, China; ^2^ Cancer Center, Shanxi Bethune Hospital, Shanxi Academy of Medical Sciences, Third Hospital of Shanxi Medical University, Tongji Shanxi Hospital, Taiyuan, China; ^3^ Shanxi Provincial Key Laboratory for Translational Nuclear Medicine and Precision Protection, Taiyuan, China

**Keywords:** head and neck malignancies, oral stents, 3D printing, radiation dose, radiotherapy

## Abstract

**Purpose:**

To compare the 3D-printed oral stents (3D-OS) and personalised hand-made stents in dentistry (DentStent) for head and neck malignancies.

**Methods:**

Twelve patients with head and neck malignant tumours in the Cancer Centre of Shanxi Bethune Hospital from 1 August 2023 to 31 September 2024 were admitted to the study. After obtaining informed consent from the patients, 3D-OS and DentStent were designed for each patient, respectively, and the patients wore them to produce two groups of radiotherapy plans. The aim was to compare the homogeneity index (HI) and conformity index (CI) of the target areas, as well as the dose differences to the organs at risk (OAR) between the two plans. Additionally, the satisfaction of the patients with the stents after use was assessed.

**Results:**

The difference in HI, CI and OAR dose to the target area between the two groups was slight, but 3D-OS took less time (∼4 h) to fabricate and resulted in higher resolution and patient satisfaction.

**Conclusion:**

The proposed 3D-OS could obtain good HI and CI in radiotherapy for head and neck malignancies, and is a new method for rapid and satisfactory personalised oral stent.

## 1 Introduction

Head and neck cancers are prevalent malignancies, and radiotherapy plays a crucial role in their management (Siegel and Giaquinto, 2024). About 50%–70% of these cancers require radiotherapy at some point during their therapeutic course. Radiotherapy not only aims to eradicate the cancer but also strives to maintain the function of the affected organs and minimize damage to healthy tissues (Peng et al., 2020). As a result, it contributes to enhancing the quality of life for patients. Despite the clinical use of high-precision radiation therapies such as IMRT, VMAT, and TOMO (Ono et al., 2024; Wang et al., 2020), which have improved the 5-year survival rate to over 80% (Trotti et al., 2003), adverse reactions associated with radiotherapy are still prevalent in head and neck tumors, including nearly universal xerostomia and a high incidence of radiation-induced oral mucositis exceeding 80% (Zaid et al., 2019). Consequently, there is a clinical need for a practical method that enhances the precision of radiotherapy and mitigates oral-related side effects during head and neck treatments.

Distance protection is one of the effective methods of radiation protection (Islam et al., 2023). Oral stents serve to efficiently isolate healthy tissues from the tumor target (Mota et al., 2023). For instance, research by Yang et al. has demonstrated that the incorporation of oral stents in the radiotherapy of head and neck cancers can notably decrease the tongue’s radiation exposure and the frequency of tongue mucositis, as well as safeguard the sense of taste (Yang et al., 2023). While tools such as oral bottles, corks, tongue depressors, and syringes have been utilized clinically to stabilize the tongue, these rudimentary devices often fail to guarantee consistent positioning of the jaw and tongue, offer limited comfort, and lack the capability for personalized fabrication (Ma et al., 2023). As a result, they fall short in fulfilling the demands of precise radiation therapy.

As precision radiotherapy advances, patients are increasingly seeking a higher standard of living. To meet this demand, researchers have recently turned their attention to personalized oral stents to enhance treatment accuracy (Ochandiano et al., 2021). The ideal oral support should combine minimal thickness and weight (typically a few millimeters thick) with sufficient structural strength, thereby minimizing space occupation within the oral cavity. Furthermore, it must accurately conform to the patient’s oral anatomy (e.g., upper and lower dental arches, tongue, etc.), ensuring the stability of oral structures during radiotherapy. This precision enhances the repeatability of target area positioning and normal tissue alignment, ultimately improving the accuracy and effectiveness of radiotherapy. In the field of dentistry, traditional methods such as gypsum impressions are used to create custom oral stents. Moreover, 3D printing technology, known for its personalized and precise fabrication, particularly suits medical applications (Wilke et al., 2017; Zhang et al., 2019). The current technical workflow for 3D-printed oral stents typically relies on CT scans, plaster model scans, or intraoral scans to capture patient-specific dentition and jawbone data. The acquired data are then processed using specialized software (e.g., Meshmixer or Rhinoceros 3D) for 3D modeling and optimization, followed by printing with photocurable resin materials (Mota et al., 2023). Digital Light Processing (DLP), a layer-by-layer printing technology, is favored for its fast printing speed and high precision (Li et al., 2019; Patel et al., 2017). Besides, Oral scans of dentition exhibit high dimensional accuracy and morphological accuracy. However, there is a scarcity of literature combining these technologies to produce oral stents.

This study introduces an innovative technique for fabricating oral stents using DLP and oral laser scanning. We aim to compare the target area dose and the dose to organs at risk (OARs) of the 3D-printed oral stents (3D-OS) with the personalised hand-made stents in dentistry (DentStent). The objective is to investigate new methods to make new oral stents for patients with head and neck malignancies. We hypothesize that the 3D-OS will demonstrate equivalent dosimetric performance (target coverage and OAR sparing) to DentStent, while significantly reducing production time and improving patient comfort.

## 2 Materials and methods

### 2.1 Clinical data

Inclusion criteria for study enrollment: patients with head and neck malignant tumors who have been pathologically diagnosed and need to receive radiotherapy (those who have not received radiotherapy before); the subjects are fully informed of the research content, voluntarily sign the informed consent form, and are able to cooperate with the stent preparation process; the age range is between 18 and 75 years old; they have the basic ability to take care of themselves; non-pregnant or lactating women; the mouth opening degree is ≥20 mm.

Twelve patients with head and neck malignancies admitted to the Cancer Center of Shanxi Bethune Hospital from 1 August 2023 to 31 September 2024 were included in the study and the patient information was shown in Table 1. DentStent and 3D-OS were designed for each patient, respectively, and the patients wore them to produce two groups of radiotherapy plans. This study was approved by the Shanxi Baithune Hospital Medical Ethics Committee for approval (Ethical review approval number: YXLL-2023-152).

**TABLE 1:** Demographics of patients.

Patient	Age	Sex	Tumor location	TNM stage	Radiation dose (Gy)/Fraction(f)
Pt1	47	Male	Oropharynx	T3N2M0	PGTV: 69.96 Gy/33f PTV: 60.06 Gy/33f
Pt2	56	Female	Hard palate	T3N0M0	PGTV: 69.96 Gy/33f PTV: 60.06 Gy/33f
Pt3	65	Male	Oropharynx	T4N0M0	PGTV: 60 Gy/30f PTV: 54 Gy/30f
Pt4	74	Male	Gingival cancer	T3N0M0	PGTV: 66 Gy/33f PTV: 59.4 Gy/33f
Pt5	60	Male	Oropharynx	T3N0M0	PGTV: 66 Gy/33f PTV: 59.4 Gy/33f
Pt6	73	Male	Oropharynx	T3N0M0	PGTV: 66 Gy/33f PTV: 59.4 Gy/33f
Pt7	49	Female	Oropharynx	T2N0M0	PGTV: 60 Gy/30f PTV:54 Gy/30f
Pt8	54	Female	Oropharynx	T3N0M0	PGTV: 66 Gy/33f PTV: 59.4 Gy/33f
Pt9	63	Female	Oropharynx	T3N0M0	PGTV: 66 Gy/33f PTV: 59.4 Gy/33f
Pt10	68	Male	Oropharynx	T2N0M0	PGTV: 60 Gy/30f PTV: 54 Gy/30f
Pt11	67	Female	Oropharynx	T3N0M0	PGTV: 66Gy/33f PTV: 59.4Gy/33f
Pt12	75	Female	Hard palate	T2N0M0	PGTV: 66 Gy/33f PTV: 59.4 Gy/33f

### 2.2 Fabrication of individualized oral stents

#### 2.2.1 The process of DentStent preparation

**FIGURE 1:**
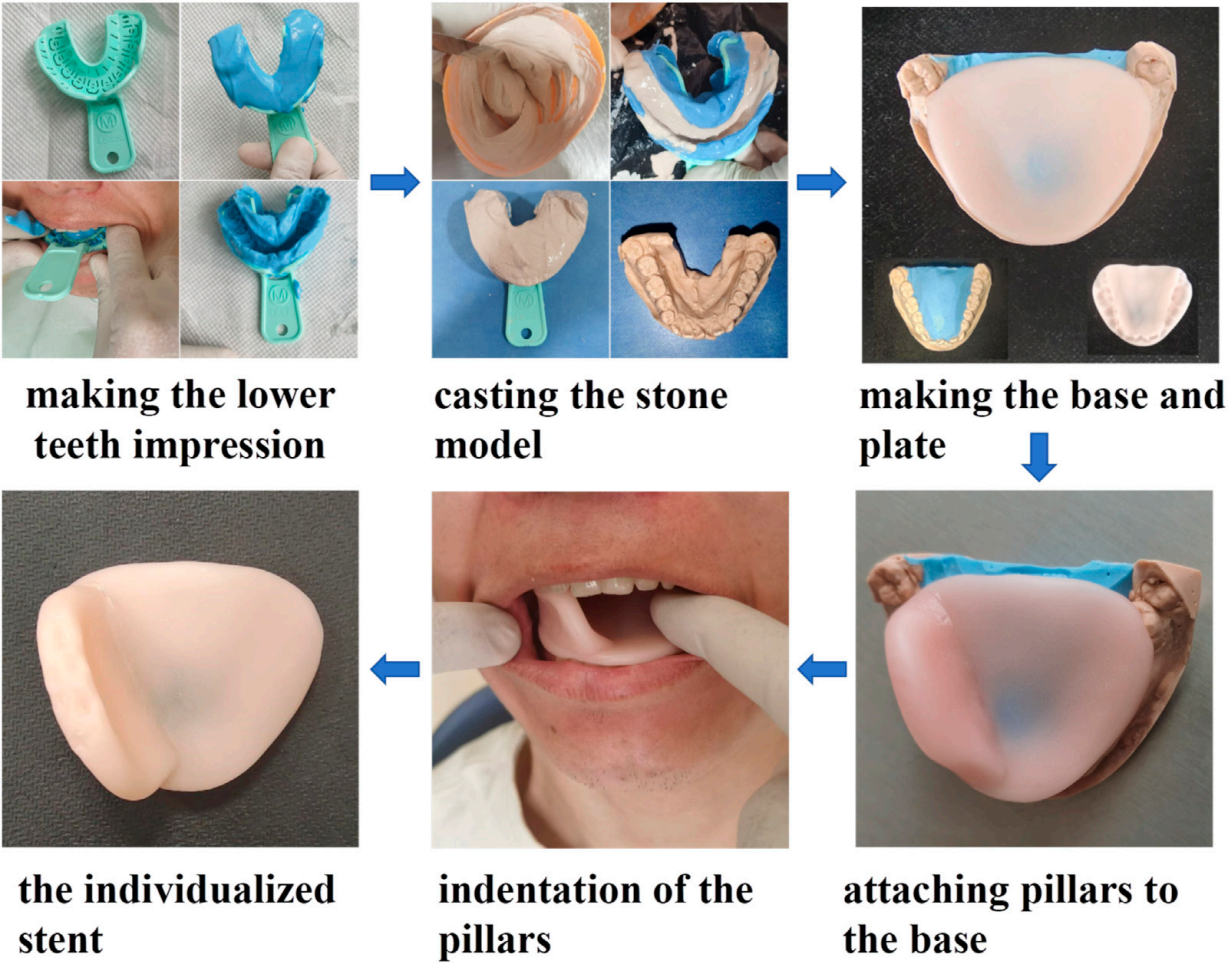
The design of DentStent.

The DentStent were made in dental department of Shanxi Bethune Hospital (shown in Figure 1).

Initially, a viscous alginate mixture was prepared by combining it with warm water and then transferred into a disposable, multifunctional dental impression tray. The patient was instructed to hold the tray in their mouth with their mouth open for about a minute before it was gently extracted. Next, plaster paste was carefully poured into the resulting dental impression, which was then left to harden at room temperature until it formed a solid stone cast. Once solidified, the stone mold was retrieved and refined using a sanding machine to eliminate any rough edges. Following this, the stone mold was filled with a fresh alginate mixture. On top of this, a tongue depressor was constructed using a blended resin (OSTRON II, from Japan), prepared precisely as per the manufacturer’s guidelines. In the final step, a layer of the resin was applied to the edge of the tongue depressor. Before the resin fully set, the tongue depressor was placed into the patient’s mouth and adjusted to ensure perfect occlusion with the lower teeth. The patient was then asked to bite down on the depressor for 20 s. Once the resin had hardened, the stent was removed from the mouth, completing the creation of the DentStent.

#### 2.2.2 The process of 3D-OS fabrication

**FIGURE 2:**
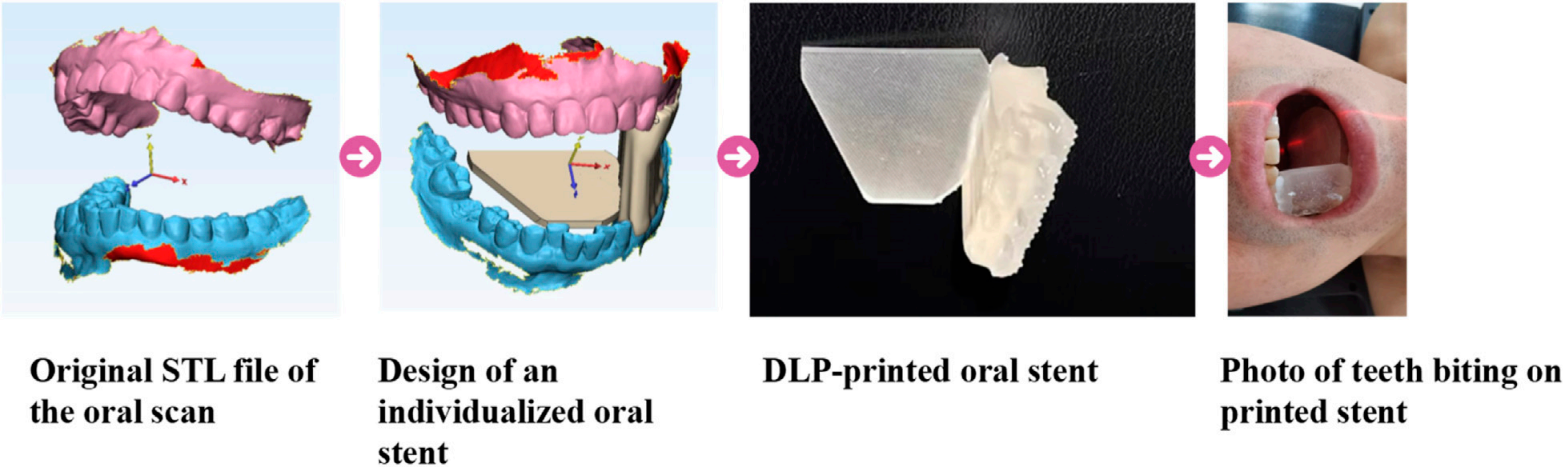
The design of oral stent by DLP printing.

The preparation steps were as follows (Figure 2): Firstly, the patient’s teeth were separated to about 2 cm apart with spacers, and the digital model (.stl) of the dentition and the fixed occlusal relationship were obtained by an oral scanner (model: 3shape, S1P-2); Secondly, in DentalDB software (version: 2.3), we select the lower dentition on the complete side and the outline is formed by connecting the midpoint of the tooth surfaces, then a 2 mm thickening is added outward to form the dental appliance. The crown surface of the dental appliance is selected and stretched to cover the midpoint of the upper tooth surface to obtain the support model. Subsequently, a semi-elliptical tongue depressor (thickness: 1 mm) was integrated into the model through a Boolean addition, resulting in the final oral stent. The designed individualized model was sent to a DLP printer (model: Shape 1 + 300). The print settings were 50 μm per layer, 50 s for the bottom layer and 5 s for the remaining layers.

In this study, we used a biocompatible transparent dental model resin (Shenzhen PioCreat 3D Technology Co. Ltd.), the composition of which is polyacrylate.

### 2.3 Design of treatment plans

For radiotherapy setup, the patient was fitted with a DentStent and 3D-OS respectively. They were then positioned supine on a CT simulator, using a U-shaped headrest along with a thermoplastic mask to immobilize the head, neck, and shoulders. The CT scan covered an area extending from 2 cm above the skull to the subclavian bone, with the slice thickness of 3 mm. The CT images were subsequently transferred to the MIM Maestro (version: 6.7.5) contouring workstation for further processing.

Combining the patient’s CT localization images, head and neck MRI images, lymph node ultrasound, pathological examination and other data, and referring to the International Commission Radiological Units (ICRU) reports No. 50, No. 62, and No. 83, the same radiotherapist will outline the gross tumor volume (GTV), clinical target volume (CTV), planning target volume (PTV) and OARs (Monti et al., 1995; Hodapp, 2012; Stroom and Heijmen, 2002). OARs mainly include the spinal cord, bilateral parotid glands, oral cavity (OC).

In the development of the radiotherapy plan, a senior physicist utilized the TOMO treatment planning system to create treatment plans. The prescribed dose was shown in Table 1. For the protection of OARs, parotid gland sparing was achieved by limiting the V_30_ (the volume receiving 30 Gy) to <50% of total gland volume. The oral cavity’s mean dose was not to exceed 35 Gy. For the eyes, the maximum dose to the lens (Dmax) was restricted to below 9 Gy. Additionally, the spinal cord’s maximum dose was not to surpass 45 Gy, and the brainstem’s maximum dose was limited to 54 Gy to ensure safety and minimize side effects.

### 2.4 Observation index

Two indexes, homogeneity index (HI) and conformity index (CI), were used to evaluate the dose in the target area. HI quantifies the uniformity of dose distribution within the target volume, where a lower HI value indicates more homogeneous dose coverage. CI evaluates the spatial congruence between the prescribed isodose volume and the planning target volume (PTV), with CI values ranging from 0 to 1. Higher CI values demonstrate superior geometric matching between the high-dose region and the target morphology. Dmean represents the average radiation dose received by the target area or surrounding normal tissues, while Dmax indicates the maximum radiation dose in that region. Dmean and Dmax were used to compare the difference of OARs under different plans. The patient’s satisfaction with the two stents in terms of the production time, the production process, the waiting time and the comfort of use was also evaluated. The fabrication timeline was systematically recorded for both methods: (1) For traditional stents, the process included impression taking, model casting, and manual stent fabrication. (2) For 3D-OS, the workflow comprised intraoral scanning, digital design, and 3D printing. The patient satisfaction evaluation was conducted through standardized oral interviews during the follow-up visit after 1 week of stent use. Answers were documented as binary outcomes (Satisfied/Unsatisfied).

### 2.5 Statistical methods

Statistical analyses were performed using SPSS software (version 23.0). Continuous data are presented as mean ± standard deviation (***x* ®** ± s). A *P*-value < 0.05 was considered statistically significant.

## 3 Results

### 3.1 Geometry in the shape of the tongue and mouth with the oral stent

**FIGURE 3:**
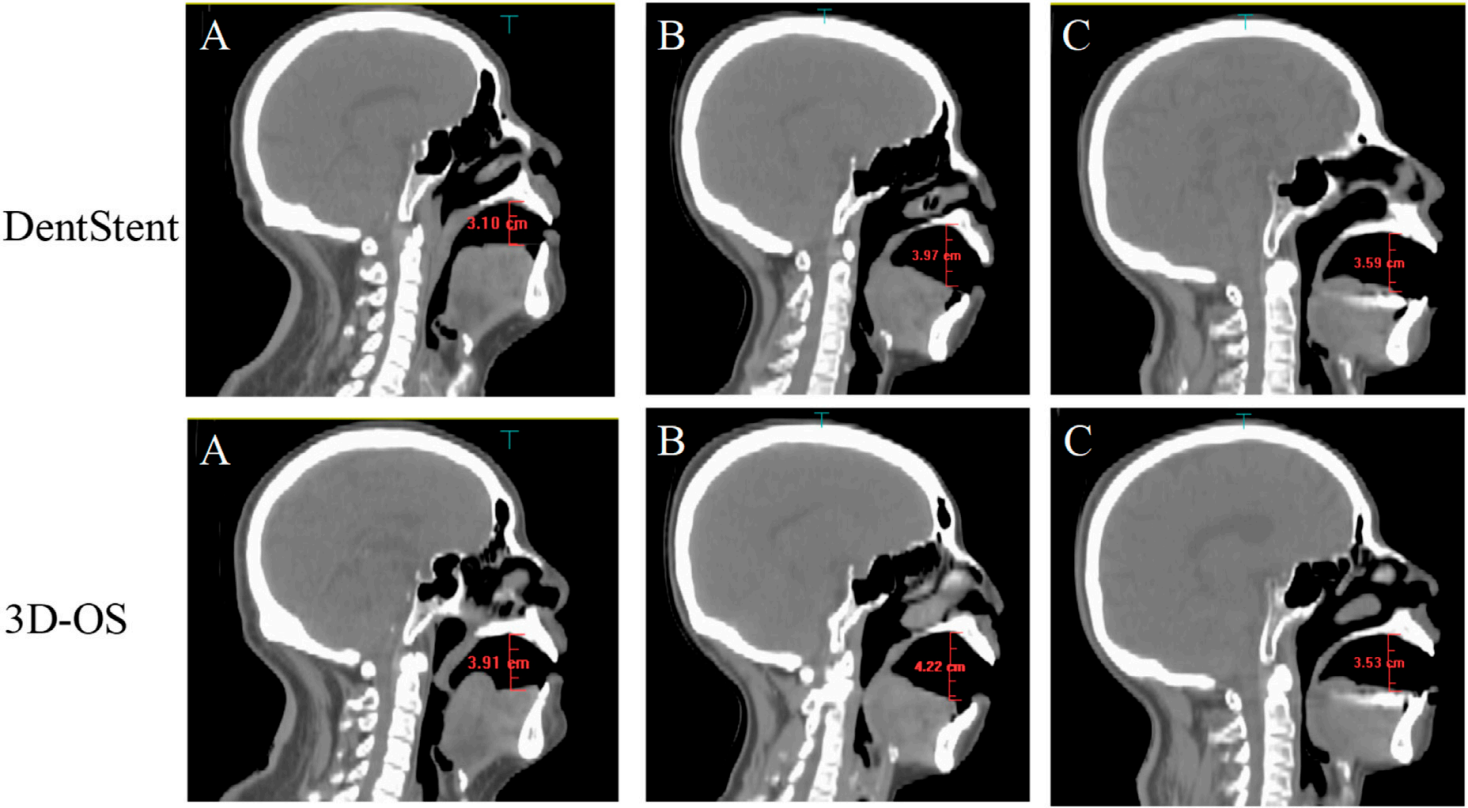
The first row shows the sagittal images of three randomly selected patients **(A, B, and C)** after using DentStent. The second row shows the sagittal images of the same three patients **(A, B, and C)** after using 3D-OS.

As shown in Figure 3, the volume and geometry of the OC changed after wearing the personalized oral stent. The average volume of the OC was 60.26.± 10.31 cm^3^ and 59.74 ± 11.34 cm^3^ for the DentStent and the 3D-OS, respectively. The different OC shapes also resulted in different distances between the dorsal surface of the tongue and the hard palate, and the distances between the dorsal surface of the tongue and the hard palate for the DentStent and the 3D-OS were 3.72 ± 0.49 cm and 3.69 ± 0.37 cm, respectively.

### 3.2 Dosimetric comparison of target volume and OARs

**FIGURE 4:**
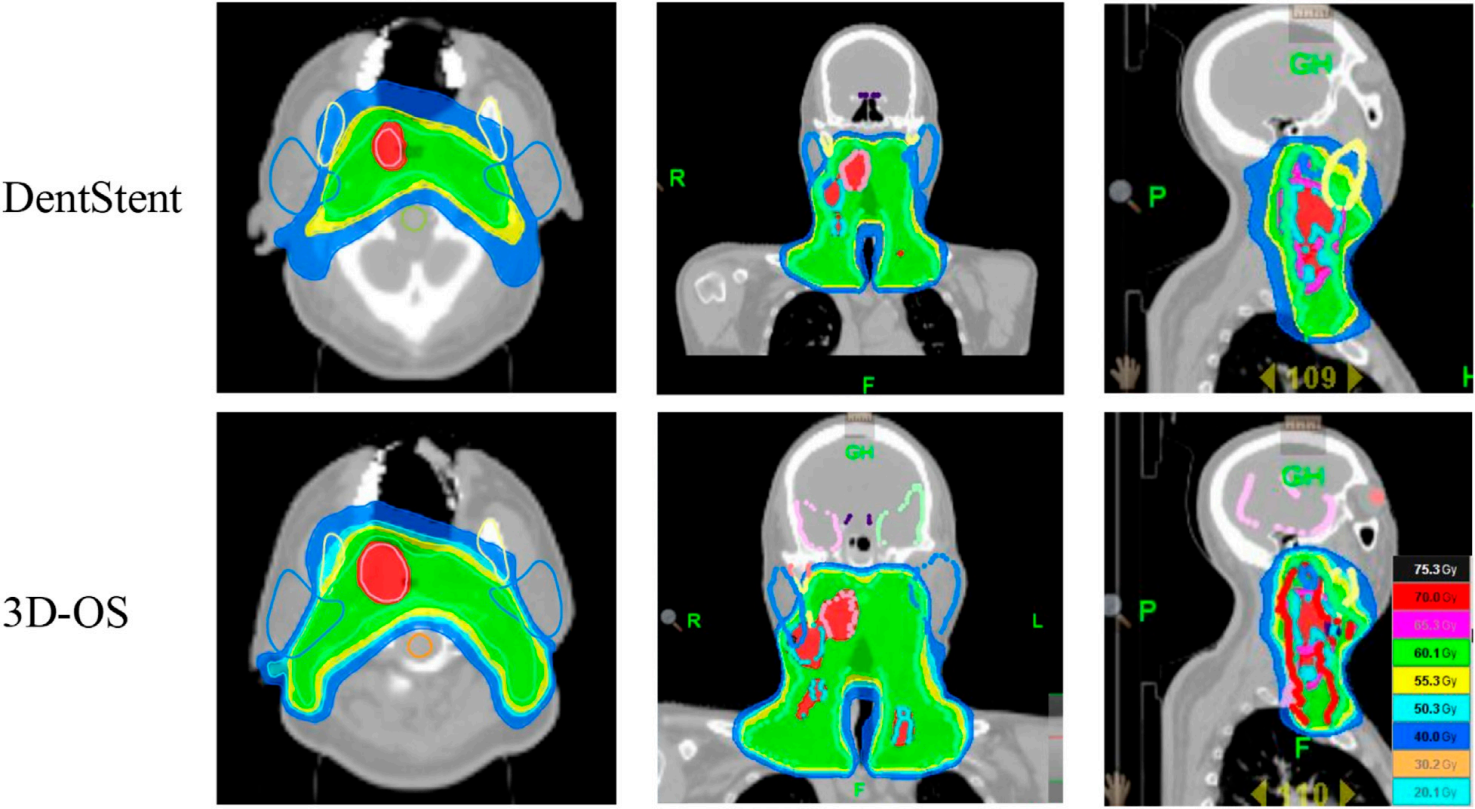
The first row shows the isodose line of one typical patient after using DentStent, and the second row shows the isodose line of the same patient after using 3D-OS.

After wearing different stents, we completed the treatment plan with the same plan settings and constraints on both CT slices and performed dosimetric comparisons. The distribution of isodose curves for the two planned groups is shown in Figure 4. Both stent types demonstrate comparable dose distribution patterns. Which are within acceptable clinical dose requirement for both groups. This finding is critical as it suggests that 3D-OS can be considered as a alternative to conventional DentStent in head and neck radiotherapy. As shown in Table 2, the mean HI of the 3D-OS was 0.14, and the mean CI was 0.77; the mean HI of the DentStent was 0.13, and the mean CI was 0.76. The dosimetric distribution of OARs was shown in Table 3. There were no statistical differences in the dose indicators of different OARs between the 3D-OS group and the DentStent group, including the maximum spinal cord irradiation dose (37.8 ± 2.5 Gy vs*.* 37.7 ± 2.1 Gy, *t* = 0.15, *P* = 0.84), the average oral cavity irradiation dose (33.31 ± 2.1 Gy vs*.* 34.72 ± 3.2 Gy, *t* = −1.82, *P* = 0.15), the average mandibular bone irradiation dose (38.3 ± 2.4 Gy vs*.* 38.1 ± 1.7 Gy, *t* = 0.33, *P* = 0.72), the average left parotid gland irradiation dose (32.8 ± 3.9 Gy vs*.* 34.5 ± 2.3 Gy, *t* = 1.84, *P* = 0.16), and the average right parotid gland irradiation dose (35.8 ± 0.6 Gy vs*.* 36.2 ± 0.7 Gy, *t* = −2.12, *P* = 0.07) (*P* > 0.05).

**TABLE 2:** Dosimetic comparison of PTV between the two plans with different stents.

Group	CI	HI	D_95%_ (Gy)	D_98%_ (Gy)	D_2%_ (Gy)
3D-OS	0.77 ± 0.03	0.14 ± 0.02	62.2 ± 0.3	60.5 ± 0.8	74.9 ± 1.5
DentStent	0.76 ± 0.04	0.13 ± 0.01	61.9 ± 0.8	60.7 ± 1.1	74.2 ± 1.3
*t*	0.98	2.19	1.72	−0.72	1.73
*P*	0.34	0.07	0.13	0.69	0.16

**TABLE 3:** Dosimetic comparison of OARs between the two plans with different stents.

Group	Cord Dmax (Gy)	Oral cavity Dmean (Gy)	Mandible Dmean (Gy)	Parotid L		Parotid R	
				Dmean (Gy)	V_30%_ (%)	Dmean (Gy)	V_30%_ (%)
3D-OS	37.8 ± 2.5	33.31 ± 2.1	38.3 ± 2.4	32.8 ± 3.9	44.3 ± 7.8	35.8 ± 0.6	49.6 ± 3.2
DentStent	37.7 ± 2.1	34.72 ± 3.2	38.1 ± 1.7	34.5 ± 2.3	42.1 ± 10.7	36.2 ± 0.7	50 ± 2.9
*t*	0.15	−1.82	0.33	1.84	0.82	−2.12	0.45
*P*	0.84	0.15	0.72	0.16	0.45	0.07	0.62

### 3.3 Subjective feedback from patients

There are a large number of patients in the dentistry clinic, so patients have a long waiting time to make the handmade stents. Dentstent fabrication requires two clinical visits. However, the duration for 3D scanning each patient is no more than 5 min, and the entire production process takes no longer than 5 h (as shown in Table 4). This efficiency gain is particularly valuable for high-volume clinics, where reduced waiting time may directly translate into improved patient satisfaction.

**TABLE 4:** Subjective feedback from patients.

Group	Number of appointments	Production time (h)	Comfort of use
3D-OS	1	4.5 ± 0.5	Yes
DentStent	2	25.2 ± 2.4	Yes

## 4 Discussion

Radiation-induced toxicities remain a significant challenge in head and neck cancer radiotherapy. At present, there is no satisfactory treatment for radiation-related adverse reactions, and it is necessary to take preventive measures to reduce the occurrence of adverse reactions (Man et al., 2024). Oral stents, as radiotherapy positioning devices, have been internationally adopted to improve treatment reproducibility and reduce radiation-induced toxicities in head and neck cancer radiotherapy (Cleland et al., 2021). In this study, we explored the novel 3D-printed oral scaffold process and compared the effects of 3D-OS and routine DentStent on the OARs, target area dose, and patient satisfaction. The results revealed that 3D-OS fabricated based on the patient’s oral structure showed comparable clinical fit to DentStent, with both devices demonstrating proper adaptation to the oral anatomy during preliminary clinical evaluations. However, the 3D printing method offers the advantages of reduced production time and increased patient contentment.

Traditional oral stents, such as those fabricated from glass vials, corks, or syringes, are cost-effective and simple to produce but suffer from poor reproducibility, displacement risks, and safety concerns (Ma et al., 2023; Feng et al., 2019; Nguyen et al., 2018). Therefore, in order to improve the accuracy and effectiveness of oral stents, it is necessary to find ways to personalize their production. When DentStent are fabricated, the workflow is often challenged by multiple appointments, intensive labor, time, experience and multidisciplinary collaboration. In addition, going through the “impression-plaster-stent” process may lead to loss of accuracy information. Compared with the handmade method, oral stents based on CT scanning are mainly affected by the accuracy of scanning images. The anatomical models obtained from CT scans have relatively large voxel sizes. This large voxel dimension can compromise the accuracy of dental occlusal surface representation, negatively affecting the delineation and reconstruction of maxillary and mandibular dental arches, ultimately resulting in reduced precision during the fabrication of oral stents. For example, Zaid et al. used diagnostic CT images to design and prepare 3D-OS with the drawbacks of no high scan quality, artifacts, subjectivity in depicting tooth anatomy, and inaccuracy in occlusal localization, which resulted in inaccurate and poorly fitted oral scaffolds (Zaid et al., 2019). Furthermore, 3D scanning-based methods overcome challenges associated with anatomical and pathological defects, such as edentulous patients and patients with severe malocclusions (Sasaki et al., 2019; Zaid et al., 2019). 3D scanning demonstrates a high level of precision, with oral scanning capable of achieving an accuracy of 50 μm (Flügge et al., 2018), which surpasses the 1 mm (Brenner and Hall, 2007) accuracy typically associated with CT scanning.

Compared with, 3D printing technology has many advantages such as improving manufacturing precision, simplifying the production processes, saving costs and human resources, shortening production time, and realizing fast personalized production (Ding et al., 2017). The digital workflow (intraoral scanning → 3D modeling → printing) used in the study reduces material waste and technician time, potentially lowering production costs. The printed oral stent features a smooth surface that facilitates cleaning, and its straightforward application process makes it convenient for patients to independently put on during radiation therapy. After use, the tongue can be pushed away from the target area, reducing the dose and volume of the tongue to be irradiated. In addition, the upper and lower surface shells of the 3D-printed oral stent have upper and lower dentition indentation grooves respectively, which avoids the movement of the oral stent after it is worn to the patient’s mouth, and ensures a good positional repeatability of the stent during the radiotherapy process. Currently, some new 3D printing materials are also widely used in dentistry, including metals, polymers, ceramics, and bioactive materials, etc. Kouji Katsura et al. found that the material of the oral stent may affect the dose distribution, as the presence of dental alloys leads to an increase in the mucosal dose due to backscattered radiation during external-beam radiation therapy (Katsura and Tanabe, 2023). This designed personalized 3D-OS was fabricated from commercial biocompatible photocurable resin that demonstrates outstanding physicochemical properties and maintains volumetric and morphological stability in the oral environment. However, this study did not evaluate the resin’s long-term biocompatibility under repeated radiotherapy sessions, an important aspect that will be addressed in future research.

The process designed in this study required about 5 h to prepare the personalized 3D-OS, whereas Zaid et al. reported a fabrication time of 48 h for handmade scaffolds (Zaid et al., 2019), thus reducing the fabrication time is one of the main advantages of 3D-printed scaffolds. The method of 3D-OS is also fast in modeling, and oral scanning can be completed within 5 min. Ma et al. prepared the 3D printed stent by letting the patient put the softened impression paste into the patient’s mouth, then instructing the patient to bite the impression paste into a synthetic shape, then the model was removed from the mouth and scanned using a large-aperture CT scan, and the scanned CT data was used to design a 3D model with ventilation channels, which is also a more cumbersome and time-consuming process than this study (Ma et al., 2023). Furthermore, Dentstent and 3D-OS were compared from the perspective of patients’ subjective feelings, the results show 3D printing are more convenient and are suitable for clinical promotion.

The evaluation of radiotherapy plan is a key step in the process of radiotherapy. In this study, CI and HI were used to evaluate the homogeneity and conformability of the target area (Tsai et al., 2024). In this study, two groups of radiotherapy plans were analyzed by wearing two types of oral stents in the same patient, as presented in Table 2, the mean HI for 3D-OS was 0.14 with a mean CI of 0.77, whereas for DentStent, the mean HI was 0.13 and the mean CI was 0.76. No statistically significant difference was observed in the dose distribution within the target range (*P* > 0.05). This indicates that the new 3D-OS designed in this study have little effect on the dose distribution in the target area. In addition to focusing on the target-area dose distribution, the OAR dose distribution is also a very important part of evaluating the quality of the radiotherapy program. Inoue et al. designed a retrospective cohort study of 34 patients with pathologically confirmed maxillary gingival, maxillary sinus, nasal cavity and soft palate cancers, and the results of dosimetric analysis showed that the median dose to the tongue (36.2:65.4 Gy) and the Dmean (4.9:25.9 Gy) in the group wearing an oral stent were significantly lower than those in the control group (*P* < 0.05) (Inoue et al., 2020). The above results indicate that the application of oral stent in radiotherapy of head and neck malignant tumors can effectively reduce the irradiated dose to organs at risk, which provides a theoretical basis for its clinical application to reduce the occurrence of adverse reactions such as dry mouth syndrome and oropharyngeal mucosal pain. In this study, by comparing with the DentStent, there is no statistically difference in the dose distribution of vital organs such as spinal cord, larynx, and bilateral parotid glands, which indicates that the 3D printed oral cavity can also effectively protect the critical organs in the oral cavity.

This study was designed as a preliminary exploratory investigation to evaluate the feasibility and potential benefits of 3D-printed oral stents in head and neck radiotherapy. Future multicenter trials with larger cohorts should validate these findings across diverse populations (e.g., varying tumor stages or dentition statuses). To mitigate potential selection bias, explicit inclusion/exclusion criteria (e.g., exclusion of edentulous patients) must be obeyed in subsequent studies. Longitudinal assessments of adverse event reduction (e.g., xerostomia incidence) will further clarify clinical utility.

## 5 Conclusion

This study provides preliminary evidence that 3D-printed oral stents offer comparable dosimetric outcomes to conventional DentStent while demonstrating advantages in fabrication efficiency and patient satisfaction. However, the small sample size and exploratory nature of this study necessitate cautious interpretation of the results. Additionally, further investigation into the long-term biocompatibility and durability of 3D-printed materials in the radiotherapy setting is required.

## Data Availability

The original contributions presented in the study are included in the article/supplementary material, further inquiries can be directed to the corresponding authors.
